# Identify GADD45G as a potential target of 4-methoxydalbergione in treatment of liver cancer: bioinformatics analysis and in vivo experiment

**DOI:** 10.1186/s12957-023-03214-3

**Published:** 2023-10-13

**Authors:** Li-Ping Zeng, Yu-Qi Qin, Xiao-Min Lu, Zhen-Bo Feng, Xian-Lei Fang

**Affiliations:** 1 Department of Pathology, Hunan University of Medicine, 492 Jinxinan RD, Huaihua, Hunan 418000 People’s Republic of China; 2 Department of Pathology, The First Affiliated Hospital of Guangxi Medical University, 6 Shuangyong RD, Nanning, Guangxi Zhuang Autonomous Region 530021 People’s Republic of China; 3 Department of Pathology, Jiangbin Hospital of Guangxi Zhuang Autonomous Region, 85 Hedi RD, Nanning, Guangxi Zhuang Autonomous Region 530021 People’s Republic of China

**Keywords:** GADD45G, Hepatocellular carcinoma, RNA sequencing, 4-Methoxydalbergione, Xenograft

## Abstract

**Background:**

The growth arrest and DNA damage-inducible gene gamma (GADD45G), an important member of GADD45 family, has been connected to the development of certain human cancers. Our previous studies have confirmed that GADD45G expression could be upregulated by 4-methoxydalbergione (4MOD) in liver cancer cells, but its potential pathological role in hepatocellular carcinoma (HCC) has not been fully understood. This study aimed to determine potential role of GADD45G in HCC, and the effects of 4-methoxydalbergione (4MOD) on the regulation of GADD45G expression in vivo were also analyzed.

**Methods:**

Publicly available data and in-house immunohistochemistry (IHC) experiments were utilized to explore the expression profiles and clinical significance of GADD45G in HCC samples. Functional enrichment analysis based on GADD45G co-expression genes was used to excavate the molecular mechanism of GADD45G in HCC. We also conducted in vivo experiment on BALB/c nude mice to excavate the inhibitory effect of 4MOD on HCC and to evaluate the differences in the expression of GADD45G in xenograft tissues between the 4MOD-treated and untreated groups.

**Results:**

GADD45G displayed significant low expression in HCC tissues. Downregulated expression of GADD45G was positively correlated with some high risk factors in HCC patients and predicted worse prognosis of HCC patients. There was a close association of GADD45G mRNA expression and immune cells, including neutrophils, NK cells, CD8 T cells, and macrophages. Co-expressed genes of GADD45G were involved in several pathways including cell cycle, carbon metabolism, and peroxisome. 4MOD could significantly suppress the growth of HCC in vivo, and this inhibitory effect was dependent on the upregulation of GADD45G expression.

**Conclusion:**

GADD45G expression can be used as a new clinical biomarker for HCC and GADD45G may be a potential target for the anti-cancer effect of 4MOD in liver cancer.

**Supplementary Information:**

The online version contains supplementary material available at 10.1186/s12957-023-03214-3.

## Introduction

Primary liver cancer is one of the most common malignancies in the world, ranking sixth among all cancers and the third leading cause of cancer deaths [1]. Hepatocellular carcinoma (HCC), the vast majority of primary liver cancer cases, has a high incidence in several countries [2]. Currently, multiple risk factors have been confirmed to be associated with HCC, including chronic hepatitis B virus (HBV) infection, chronic hepatitis C virus (HCV) infection, aflatoxin, alcohol consumption, obesity, and diabetes, but the pathogenesis of HCC is not yet understood [3–7]. Although a variety of therapies, including surgical resection, chemotherapy, immunotherapy, and targeted therapy, have been widely used in HCC patients treatment and proved to prolong their survival, the 5-year overall survival rate of HCC patients is still unsatisfactory [8, 9]. Hence, further exploration of the molecular pathology and the identification of novel treatment approaches for HCC treatment are warranted.

The development of HCC is an extremely complex pathophysiological process, involving a series of abnormal gene expressions caused by genetic and environmental factors. Thanks to the rapid development of high-throughput sequencing technology, a large number of public datasets such as gene expression omnibus (GEO) and the cancer genome atlas (TCGA) have emerged, through which researchers can obtain a large number of experimental research data, providing important clues for the study of the pathogenesis, biomarkers, and drug targets of HCC. The growth arrest and DNA damage-inducible gene gamma (GADD45G), also known as GADD45γ, is located on human chromosome 9q22.2 with a molecular weight of 17 kD, belonging to one of the GADD45 family members [10]. Due to gene promoter methylation, GADD45G is expressed at low levels in many human tumors [11, 12]. GADD45G has been shown to participate in cell cycle arrest, cell growth, apoptosis, and DNA damage repair and play a critical role in the carcinogenesis process [12–14]. In previous study, we have confirmed that GADD45G is an effective target of 4-methoxydalbergione (4MOD) in the anti-HCC process, and the downregulation of GADD45G can reduce the inhibitory effect of 4MOD on HCC [15]. A few studies have also reported that GADD45G downregulated expression can promote HCC progression and drug resistance [16–18]. However, the expression levels, clinical significance, and potential mechanism of GADD45G in HCC have not been fully understood.

In recent years, traditional Chinese medicines have been gradually proved by modern medical technology, which have significant anti-cancer effects and have low toxic and side effects [19–21]. 4-Methoxydalbergione (4MOD), a new flavonoid isolated from the heartwood of *Dalbergia sissoo *Roxb, has been confirmed to elicit remarkable anti-cancer effects on esophageal cancer, bladder cancer, and glioma [22–24]. We have previously reported that 4MOD may inhibit the growth of HCC cells in vitro by promoting the expression of GADD45G. However, the anti-HCC effect of 4MOD in vivo and its complex molecular mechanism remain to be further explored [15].

Herein, we analyzed the expression patterns and prognostic value of GADD45G in HCC through immunohistochemistry (IHC) and bioinformatics methods. The potential pathological role of GADD45G in HCC was also investigated by analyzing the correlation with immune cells, by performing biological functions enrichment of co-expressed genes with GADD45G. In addition, the inhibitory effect of 4MOD on HCC was evaluated in nude mice. Quantitative real-time polymerase chain reaction (qRT-PCR) and IHC were used to further investigate the expression changes of GADD45G in xenograft tissues after 4MOD treatment.

## Materials and methods

### Cell culture and reagents

The human liver cancer cells SK-HEP-1 was obtained from the Procell Life Science & Technology Co., Ltd (Wuhan, China) and was authenticated through STR genotyping. The cell lines were tested periodically for mycoplasma contamination during the experiment and were confirmed negative. Cells were maintained in Dulbecco’s Modified Eagle Medium (DMEM, Gibco, CA, USA) supplemented with 10% fetal bovine serum (FBS, Gibco, CA, USA) and 1% penicillin–streptomycin solution (Beyotime, Shanghai, China).

4MOD was purchased from Shanghai Yuanye Biotechnology Co., Ltd. (Shanghai, China). In animal experiments, 0.25% polyoxyl castor oil (Yuanye, Shanghai, China) and 0.25% EtOH were used to dissolve 4MOD. A rabbit polyclonal antibody anti-GADD45G was obtained from Bioss (Beijing, China).

### HCC xenografts in nude mice

Twenty-four female BALB/c nude mice, aged 6 weeks, 18–20 g, were obtained from Hunan SJA Laboratory Animal Co. Ltd. (Changsha, China). All nude mice were placed at a temperature of 24 °C, 12 h light/12 h dark, without any specific pathogen. SK-HEP-1 cells (1 × 10^7^ cells/mouse) were subcutaneously injected into the right armpit skin of each nude mouse. After 10 days of inoculation, mice were randomly divided into control group, 4MOD low-dose group, and 4MOD high-dose group when the tumors reached a diameter of 3–5 mm. Mice in the low-dose and high-dose 4MOD treatment groups were intraperitoneally injected with 10 mg/kg and 30 mg/kg of 4MOD every 3 days, respectively, while the mice in control group received normal saline. The tumor volumes were measured and calculated according to the formula: tumor volume = (short diameters × short diameters × long diameters)/2. At the end of experiment, the nude mice were euthanized, the tumor weights were measured, and the heart, spleen, liver, kidney, and lung of each mouse were collected. One part of tumor tissues were immediately preserved at − 80 °C for qRT-PCR assay. The rest of HCC xenograft tissues were fixed with 10% formaldehyde for IHC experiment. Animal experiments in this study were approved by the Animal Ethics Committee of Hunan Academy of Traditional Chinese Medicine (2022–0025) and in accordance with the relevant ethical regulations.

### qRT-PCR

Total RNA in transplanted tumor tissues was extracted using RNAeasy Animal RNA Isolation Kit (Beyotime, Shanghai, China). The purity and concentration of total RNA were measured by NanoDrop 2000 (Thermo Scientific, USA). SureScriptTM First-Strand cDNA Synthesis Kit (GeneCopoeia, Beijing, China) was used to reverse transcribe cDNA from 1 µg of extracted total RNA according to manufacturer’s instructions. SYBR Green Fast qPCR Kit (ABclona, Wuhan, China) was used in this study for performing qRT-PCR process with the LightCycler® 480 (Roche) system. The forward primer sequence of GADD45G was 5′-CTACGAGTCCGCCAAAGTCC-3′ (Gensys Biotech, Nanning, China), and the reverse primer sequence was 5′-TTCTCACAGCAGAACGCCTG-3′ (Gensys Biotech, Nanning, China). The forward primer sequence of GAPDH was 5′-GGTTGTCTCCTGCGACTTCA-3′ (Sangon Biotech, Shanghai, China), and the reverse primer sequence was 5′-TGGTCCAGGGTTTCTTACTCC-3′ (Sangon Biotech, Shanghai, China). Relative expression of GADD45G mRNA in control and 4MOD-treated HCC xenograft tissues were calculated by the 2-ΔΔCt method.

### IHC

Seventy-six HCC and 60 paracancerous tissues were collected from the First Affiliated Hospital of Guangxi Medical University between 1 April 2019 and 1 May 2021. 4MOD-treated and 4MOD-untreated tumor tissues were obtained from the aforementioned HCC nude mouse xenografts. The primary antibody used in IHC assay was a rabbit polyclonal antibody anti-GADD45G (1:200 dilution, Bioss, Beijing, China). The secondary antibody kit (PK1006, Proteintech, Wuhan, China) was purchased from Wuhan Sanying Biotechnology. After formalin fixation and paraffin embedding, all tissues were cut into 4-μm sections, and then deparaffinized and rehydrated. Pressure cooking with Tris–EDTA buffer (PH 9.0) for 5 min was used for antigen retrieval, and then the sections were blocked with 3% H_2_O_2_ and 10% normal goat serum. After blocking, tissue sections were incubated with GADD45G antibody overnight at 4 °C, washed with phosphate-buffered saline (PBS), and incubated with secondary antibody at room temperature for 1 h. IHC staining score in this study were calculated using a semi-quantitative approach described in detail in previously reference [25].

### Differential expression analysis of GADD45G mRNA

RNA sequencing (RNA-seq) data for 374 liver hepatocellular carcinoma (LIHC) and 50 normal liver tissues were downloaded from TCGA database (https://portal.gdc.cancer.gov), and all the data were log2 transformed by R software. As the RNA-seq data of normal liver tissues in the TCGA database were relatively rare, we obtained additional transcriptome data of 110 normal liver tissues from the genotype-tissue expression project (GTEx) database to compare the GADD45G mRNA between HCC and normal liver tissues. Microarray data of GADD45G mRNA expression in human HCC and non-HCC samples were collected from the GEO (http://www.ncbi.nlm.nih.gov/geo/) database. The search terms in the present study were as follows: (hepatocellular OR HCC OR hepatic OR liver) AND (tumour OR tumor OR carcinoma OR cancer OR neoplas* OR malignan*). The inclusion criteria and exclusion criteria for GEO database in this study can be referenced in our previous work [26]. All microarrays were normalized in R software using the “limma” package to correct heterogeneity between HCC and non-HCC cases. Scatter plots of expression levels of GADD45G mRNA in HCC and normal liver tissues were drawn using R software “ggplot2” package. Standardized mean differences (SMD) were calculated in Stata12.0 software and a meta-analysis of GADD45G mRNA expression data was performed using random effect models. Finally, we used the Begg funnel plot in Stata12.0 to test publication bias. A *p* value > 0.05 was considered no publication bias. Next, we plotted the summarized receiver’s operating characteristics (SROC) curve based on the expression values of GADD45G and calculated the AUC of the SROC curve using Stata12.0 software. The AUC value of the SROC curve was used to evaluate the ability of GADD45G mRNA to distinguish between HCC and non-HCC tissues. An AUC value of 0.5–0.7 indicated poor distinguish ability, an AUC value of 0.7–0.9 indicated moderate distinguish ability, and an AUC value of > 0.9 indicated good distinguish ability.

### Prognostic value analysis of GADD45G mRNA in HCC based on TCGA data

Clinicopathological parameters and survival data of 374 HCC patients were collected from TCGA. The association of GADD45G mRNA expression and various clinicopathological parameters was analyzed through SPSS26.0 software. R software “survminer” and “survival” package were used for survival analysis and Kaplan–Meier (KM) survival curve drawing. Differences in overall survival (OS), disease specific survival (DSS), and progress free interval (PFI) between high GADD45G group and low GADD45G group were compared using log-rank test.

### Relationship between GADD45G expression and infiltration levels of immune cells in HCC

The association between expression of GADD45G mRNA and immune cell markers was characterized in HCC. These immune cell markers include 24 kinds of cells, such as CD8 T cells, dendritic cells (DC), activated dendritic cells (aDC), B cells, cytotoxic cells, macrophages, eosinophils, immature DC (iDC), mast cells, CD56bright NK cells, neutrophils, CD56dim NK cells, plasmacytoid DC (pDC), NK cells, T cells, T central memory cells (Tcm), T helper cells, T follicular helper cells (TFH), T effector memory cells (Tem), Th1 cells, T gamma delta cells (Tgd), Th2 cells, T regulatory cells (TReg), and Th17 cells [27]. The tumor immune cell infiltration levels were calculated using the single-sample GSEA (ssGSEA) method of R “GSVA” package. Spearman’s correlation was conducted between GADD45G and the immune cell infiltration level, and the analysis results were visualized using “ggplot2” package. *p* value < 0.05 was regarded as statistically significant.

### Gene Ontology (GO) and Kyoto Encyclopedia of Genes and Genomes (KEGG) enrichment analysis

Co-expressed genes of GADD45G were obtained from cBioPortal database (http://www.cbioportal.org/index.do). Spearman’s correlation ≥ 0.3 or ≤  − 0.3 were the screening conditions for co-expressed genes. Then, GO and KEGG analysis of co-expressed genes were conducted using “clusterProfiler” package of R software.

### Statistical analysis

SPSS v.26 software was used for statistical analysis, and R software packages v3.6.1, Stata v.12.0, and GraphPad Prism v.8.0 were used for results visualization. The *t*-test was used to compare the two group data with normal distribution. Multi-group data with normal distribution were compared using one-way ANNOVA. The Kruskal–Wallis test was used for data with non-normal distribution. A *p* value < 0.05 was considered statistically significant.

## Results

### GADD45G mRNA is lowly expressed in HCC tissues

**Fig. 1:**
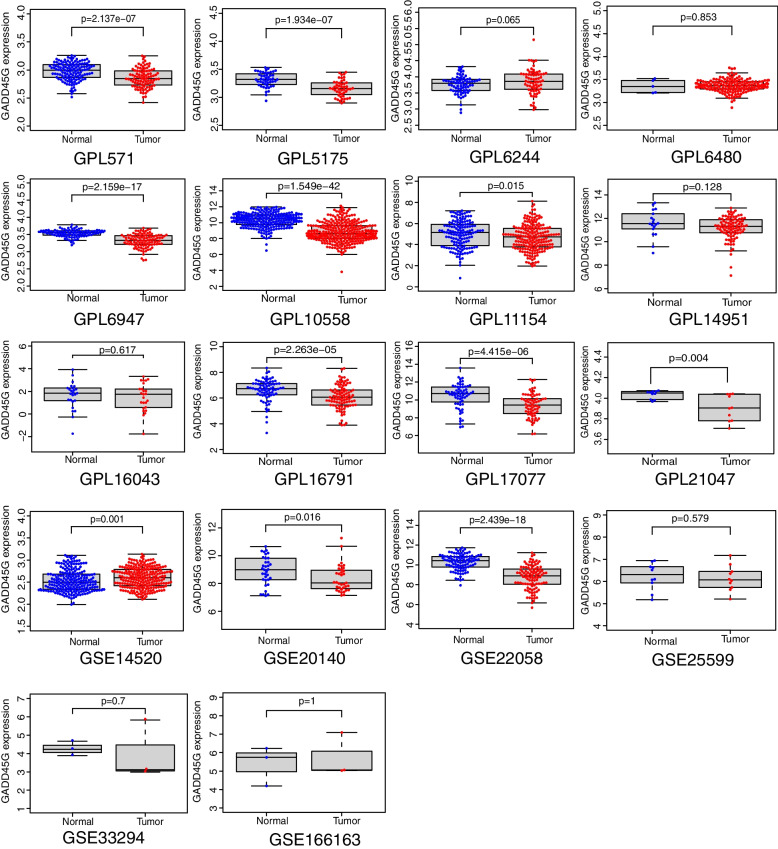
Scatter plots of GADD45G mRNA expression in HCC tissues and normal liver tissues

**Fig. 2:**
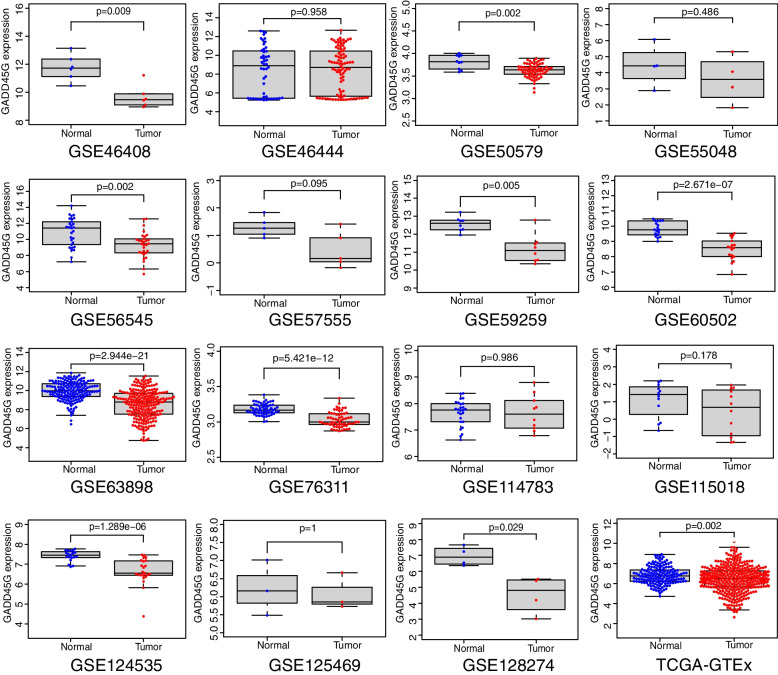
Scatter plots of GADD45G mRNA expression in HCC tissues and normal liver tissues

**Fig. 3:**
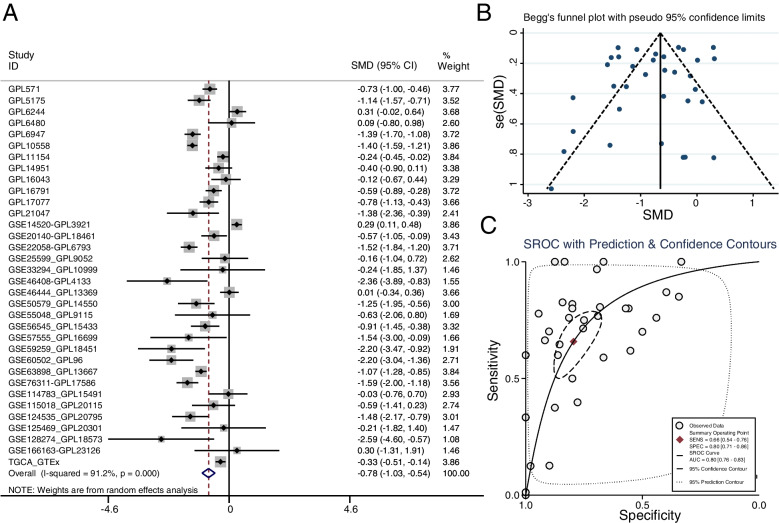
Meta-analysis of GADD45G mRNA expression in HCC and normal liver tissues. **A** The forest map of GADD45G expression levels in HCC based on RNA-seq dataset and 33 microarrays datasets; **B** publication bias tested in this study using Begg’s test; **C** SROC curve analysis of GADD45G mRNA distinguishes liver cancer from normal liver tissues

In the present study, the TCGA-GTEx RNA-seq dataset and 33 microarray datasets included 2593 HCC samples and 1875 non-HCC samples were enrolled in GADD45G mRNA differential expression analysis. Scatter plots of GADD45G mRNA expression in each dataset were shown in Figs. 1 and 2. It could be seen from scatter plots that GADD45G was lowly expressed in TCGA-GTEx and most microarray datasets. The forest map generated through merging GADD45G expression data in HCC and non-cancer liver samples from RNA-seq dataset and microarray datasets also confirmed the significant low expression of GADD45G mRNA in HCC (SMD, − 0.78; 95% CI, − 1.03 to − 0.54; *p* = 0.000) (Fig. 3A). Begg’s test showed that there was no publication bias in this study (*p* = 0.331) (Fig. 3B).

To further evaluate the discriminatory ability of GADD45G mRNA expression in HCC samples, we calculated AUC value of SROC curves of all included datasets to assess this discriminatory ability. It was found that the AUC value was 0.80 (95% CI = 0.76–0.83), suggesting that GADD45G mRNA had a moderate ability to distinguish liver cancer from normal liver tissues (Fig. 3C).

### Low expression of GADD45G mRNA indicates unfavorable prognosis in HCC patients

**Fig. 4:**
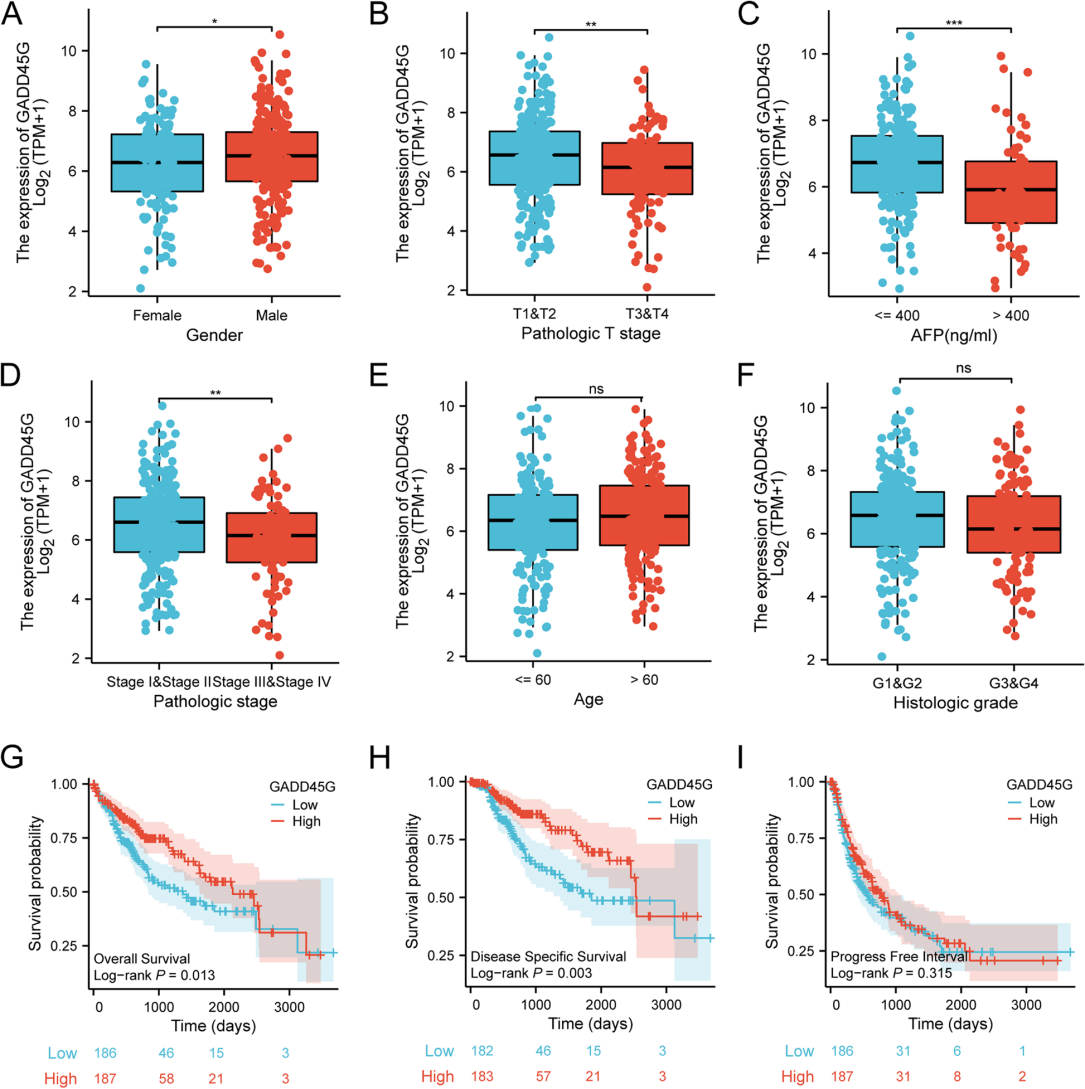
Relationship analysis between GADD45G expression and gender (**A**), pathologic T stage (**B**), AFP (**C**), pathologic stage (**D**), age (**E**), histologic grade (**F**), OS (**G**), DSS (**H**), and PFI (**I**) based on TCGA database. ns, *p* > 0.05; *p < 0.05; **p < 0.01; ***p < 0.001

Through statistical relationship analysis between GADD45G and various clinicopathological parameters based on TCGA database, we found that GADD45G mRNA expression levels were significantly correlated with HCC patients’ gender, T stage, serum AFP level, and pathologic stage. Female HCC patients had lower GADD45G mRNA expression levels than male HCC patients (*p* < 0.05) (Fig. 4A). The expression of GADD45G mRNA in HCC patients with T3 and T4 stage was lower than that in T1 and T2 stage (*p* < 0.01) (Fig. 4B). HCC patients with high serum AFP level had significantly lower GADD45G mRNA expression than those with low serum AFP level (*p* < 0.001) (Fig. 4C). The GADD45G mRNA expression in patients with pathologic stage III and IV was lower than that in stage I and II (*p* < 0.01) (Fig. 4D). However, associations could not be found between GADD45G mRNA expression and other clinicopathological parameters, including age and histologic grade (Fig. 4E–F).

The differences in OS, DSS, and PFI between the GADD45G high-expression groups and the low-expression groups were compared with log-rank test. We found that HCC patients with high GADD45G expression had longer overall survival time than those with low expression of GADD45G (*p* < 0.05) (Fig. 4G). Disease-specific survival analysis showed that patients with high GADD45G expression had better disease-specific survival time than those with low expression (*p* < 0.01) (Fig. 4H). However, the expression of GADD45G was not correlated with HCC patients’ progression-free interval survival time (*p* > 0.05) (Fig. 4I).

### GADD45G protein is lowly expressed in HCC tissues based on in-house IHC

**Fig. 5:**
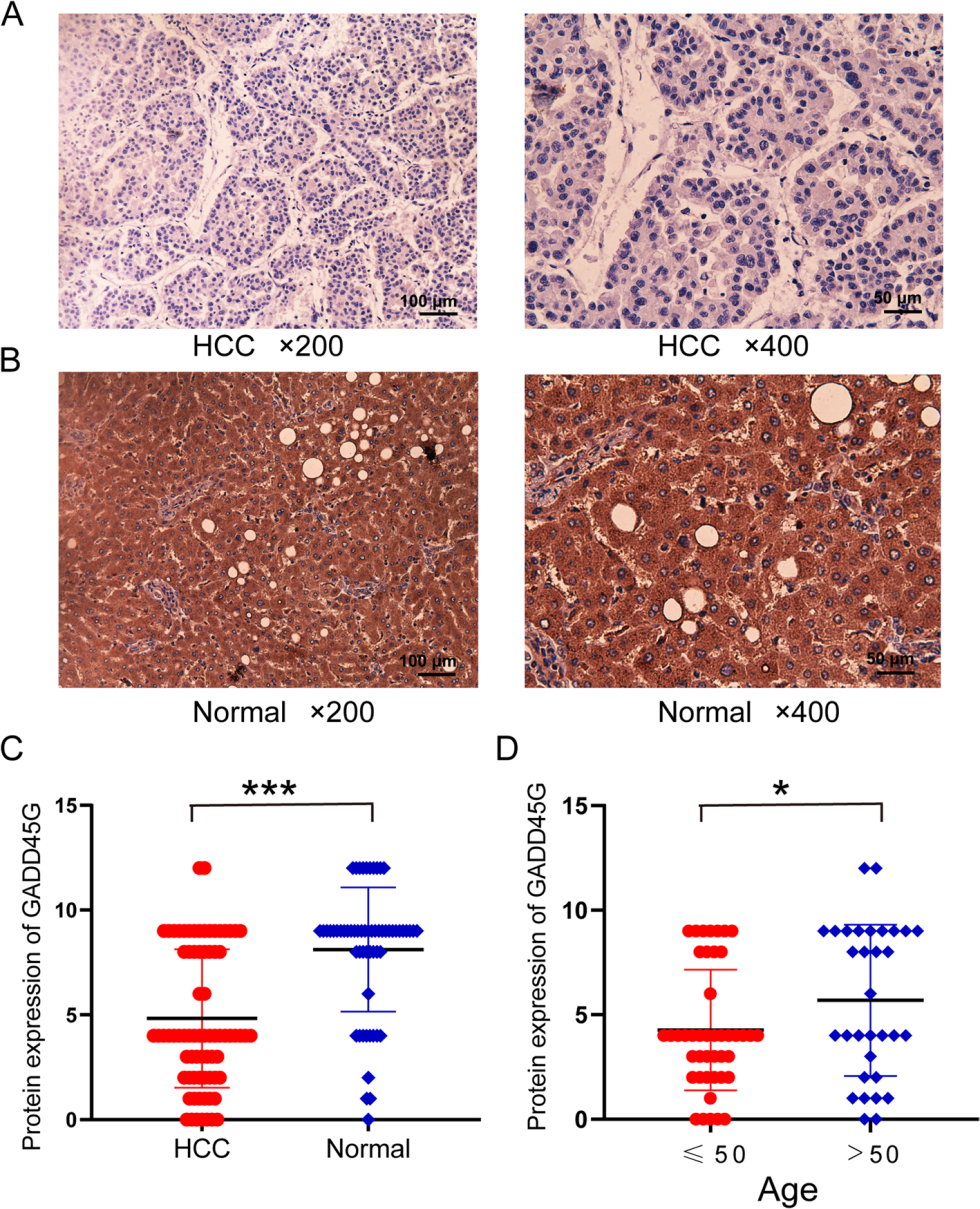
IHC staining for GADD45G protein expression in HCC and normal liver tissues. **A** The expression of GADD45G protein in HCC; **B** the expression of GADD45G protein in normal liver tissues. **C** Scatter plot of GADD45G protein expression in HCC and normal liver tissues. **D** Expression of GADD45G protein in different age groups of HCC patients. *p < 0.05; ***p < 0.001

The results of IHC assay showed the negative staining of GADD45G in HCC samples and the positive cytoplasm staining of GADD45G in non-HCC samples (Fig. 5A, B). To further quantify the IHC results, statistical differences were calculated between IHC scores in HCC and non-HCC tissues, showing that GADD45G protein was downregulated expression in HCC tissues (*p* < 0.001) (Fig. 5C). Furthermore, the relationship between GADD45G protein expression and various clinicopathological parameters, such as age, gender, TNM stage, and histological stage of HCC patients, were performed. The results revealed that there was significant difference in GADD45G protein expression related to age. The expression level of GADD45G protein in patients younger than 50 years (≤ 50 years) was lower than that in patients older than 50 years (> 50 years) (Fig. 5D). However, no significant differences were found in present study between GADD45G protein expression and gender, TNM stage, and histological stage (Table S1).

### Relationship between GADD45G expression and infiltration levels of immune cells in HCC

**Fig. 6:**
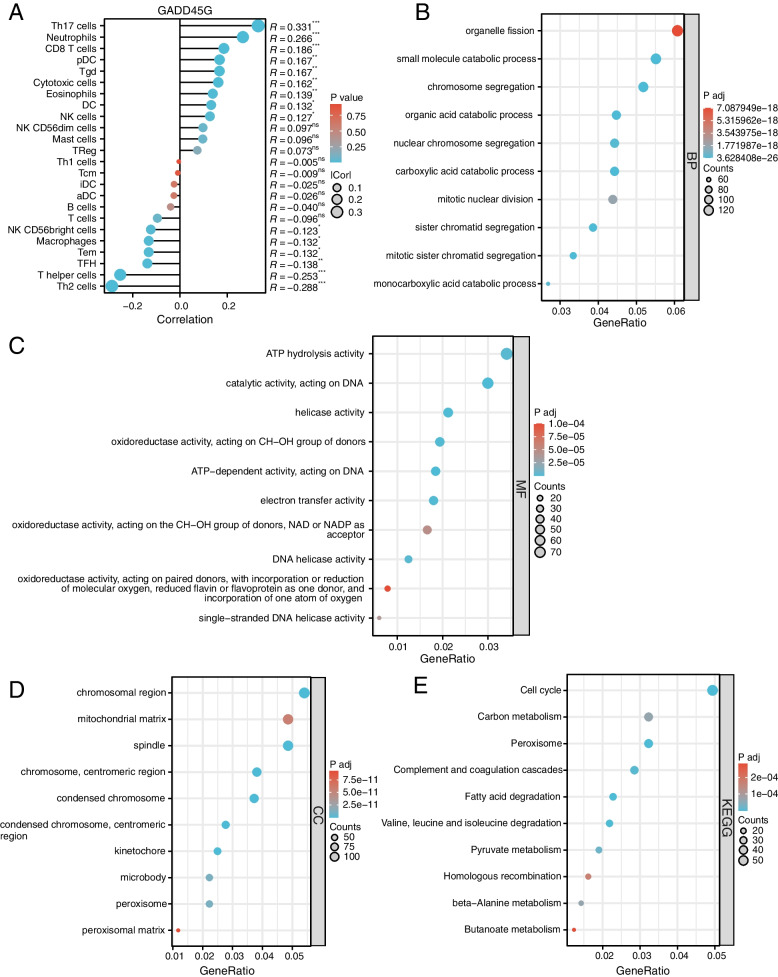
Potential molecular mechanisms analysis of GADD45G in HCC. **A** Relationship between GADD45G expression and infiltration levels of 24 kinds of immune cells in HCC. **B** GO-BP category analysis of GADD45G co-expression genes; **C** GO-MF category analysis of GADD45G co-expression genes; **D** GO-CC category analysis of GADD45G co-expression genes; **E** KEGG analysis of GADD45G co-expression genes

Immune cell infiltration is closely associated with tumor progression. Thus, we analyzed correlations between GADD45G mRNA expression and 24 kinds of immune cell markers in HCC. We found that the expression of GADD45G was positively correlated with Th17 cells, neutrophils, CD8 T cells, pDC, Tgd, cytotoxic cells, eosinophils, DC, and NK cells, while negatively correlated with NK CD56bright cells, macrophages, Tem, TFH, T helper cells, and Th2 cells (Fig. 6A).

### Potential molecular mechanisms analysis of GADD45G in HCC

In order to further explore the molecular mechanisms of GADD45G in liver cancer, we performed GO and KEGG functional enrichment analysis of 2336 co-expressed genes of GADD45G identified from cBioPortal database. Results of GO “biological process (BP)” category analysis revealed that GADD45G co-expression genes in HCC were significantly aggregated in organelle fission, small molecular catabolic process, and chromosome segregation (Fig. 6B and Table S2). Results of GO “molecular function (MF)” category analysis showed that GADD45G co-expressed genes in HCC were mainly participated in ATP hydrolysis activity, acting on DNA and helicase activity and catalytic activity (Fig. 6C and Table S3). Furthermore, GO “cellular component (CC)” category analysis results showed that co-expressed genes of GADD45G in HCC were mainly involved in chromosomal region, mitochondrial matrix, and spindle (Fig. 6D and Table S4). The results of KEGG analysis showed that co-expressed genes of GADD45G were mainly enriched in cell cycle, carbon metabolism, and peroxisome (Fig. 6E and Table S5).

### 4MOD inhibits the growth of liver cancer in vivo by promoting the expression of GADD45G

**Fig. 7:**
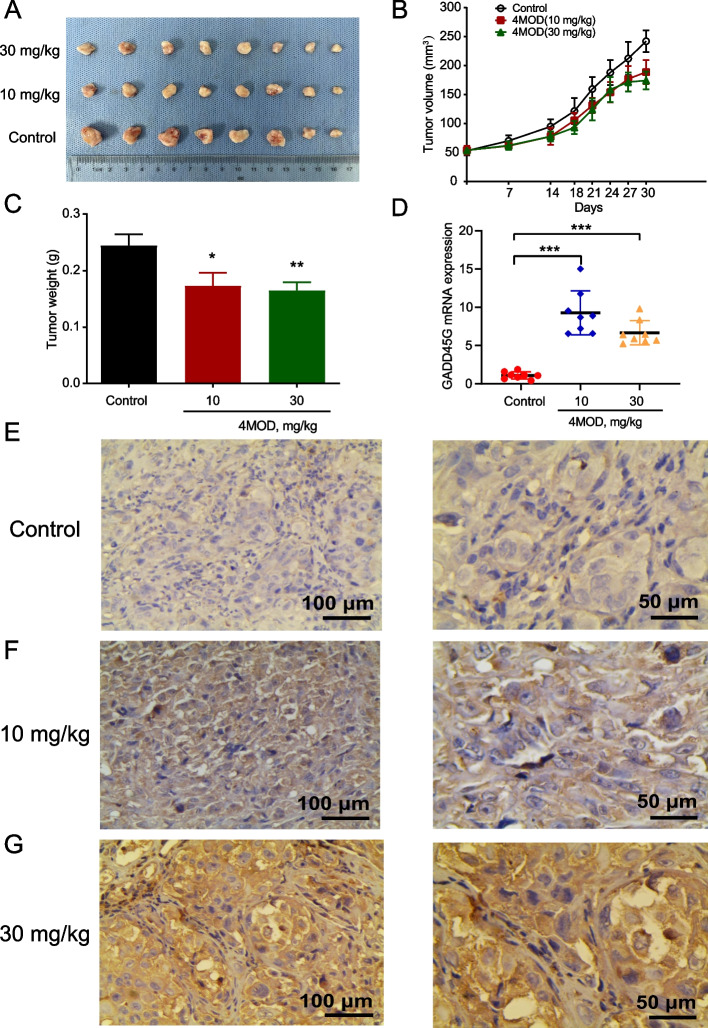
Anti-HCC effects and underlying mechanism of 4MOD in HCC xenografts. **A** Effect of different concentrations of 4MOD treatment on the tumor weight in nude mice. **B** Line chart of tumor volume change in each group; **C** histogram of tumor weight comparison in each group; **D** scatter plot of GADD45G mRNA expression in HCC xenograft tissues of different concentrations of 4MOD treatment groups and control group. **E**–**G** IHC staining for GADD45G protein expression in HCC xenograft tissues of different concentrations of 4MOD treatment groups and control group (left: × 200, right: × 400)

To ascertain the anti-HCC effects of 4MOD in vivo, HCC cells (SK-HEP-1) were injected subcutaneously to establish subcutaneous liver cancer xenograft nude mice model. Based on the measured tumor volumes, we found that tumor volumes in high-dose groups were significantly smaller than those in the control group (Fig. 7A–B and Table 1). However, no significant difference was found in tumor volumes between control groups and low-dose groups (Fig. 7A–B and Table 1). Compared with the control group, the tumor weight in each 4MOD treatment groups was significantly reduced (Fig. 7C). To further evaluate the toxic effects of 4MOD on the normal organs of mice, HE staining was performed on the major organs of mice, and the results showed that 4MOD showed no obvious toxicity to normal tissues of mice (Fig. S1).

**Table 1:** Changes of tumor volume in nude mice in each group

Group	Tumor volume (mm^3^) ($\overline{\mathrm{X}}$ ± SD)	
	Pre-treatment	Post-treatment
Control	53.1 ± 22.3	242.1 ± 52.6
Low concentration of 4MOD	54.2 ± 21.8^a^	188.9 ± 58.6^c^
How concentration of 4MOD	53.8 ± 14.0^b^	174.4 ± 43.1^d^

^a,b^*p* > 0.05 vs. control group

^c^*p* = 0.077 vs. control group

^d^*p* = 0.013 vs. control group

To reveal the molecular mechanism of 4MOD against HCC in vivo, we performed qRT-PCR and IHC assays to examine GADD45G expression in xenograft tumors. We found that GADD45G expression level was significantly increased in the 4MOD-treated mice (Fig. 7D–G). These results indicated that 4MOD suppressed the growth of HCC in vivo via promoting the expression of GADD45G.

## Discussion

The GADD45 family genes contain three members GADD45A, B, and G, encoding GADD45α, β, and γ proteins, respectively [10, 28, 29]. The GADD45 genes play an important role in cytotoxic and non-toxic stress responses, acting as stress sensor. When DNA damage, all members of the GADD45 protein family will be rapidly induced and actively participate in the DNA repair mechanism, inducing cell cycle arrest and apoptosis. Studies have indicated that GADD45G can lead to cell cycle arrest, promote cell growth, inhibit cell apoptosis, regulate DNA damage repair, and participate in the occurrence and development of tumors [10]. It has been confirmed that GADD45G is abnormally expressed in various malignant tumors, such as esophageal cancer, breast cancer, and acute myeloid leukemia [30–32]. Downregulation of GADD45G expression could enhance tumor proliferation and metastasis [11, 13]. In recent years, a number of studies have tried to clarify the relationship between GADD45G and liver cancer. A previous study reported that GADD45G mRNA expression was downregulated in liver cancer and could induce cell cycle G2/M phase arrest, thus playing a significant role in cell growth [13]. In HCC cells, downregulation of GADD45G expression could inhibit cellular senescence by activating JAK-STAT3 signaling pathway [16]. However, the specific role of GADD45G in liver cancer remains unclear.

In present study, we used microarrays and RNA-seq data to confirm that the expression level of GADD45G in HCC samples was significantly downregulated. A low expression level of GADD45G protein was further validated based on IHC staining. Our study was in line with the concept of evidence-based medicine and was the first to study the expression of GADD45G in HCC based on multi-platform datasets. In addition, SROC curve analysis showed that GADD45G had moderate ability to distinguish HCC from normal liver tissues, suggesting that GADD45G may be an important molecular marker for HCC diagnosis.

Clinicopathological parameters and survival data of HCC patients were obtained from the TCGA database to explore the prognostic value of GADD45G expression. Compared with male HCC patients, the expression level of GADD45G was lower in female patients. Low GADD45G expression was related to high tumor T stage, high pathologic stage, and high AFP levels in patients with HCC. Low expression level of GADD45G predicted shorter OS and poor DSS. These findings suggested that GADD45G may be a valuable biomarker for predicting prognosis of HCC patients, identifying high-risk patients.

Because tumor microenvironment contributes to the malignant progression of human cancers, we performed correlation analysis between GADD45G and multiple immune cells infiltration in HCC. The results showed that the expression of GADD45G was associated with a variety of immune cells infiltration, from which we conjectured that the abnormal expression of GADD45G in HCC may affect immune cells, such as memory Th17 cells, neutrophils, CD8 T cells, cytotoxic cells, and NK cells. Through enrichment analysis of GADD45G co-expressed genes, various biological processes and pathways of GADD45G involved in HCC occurrence were screened out. We found that GADD45G was associated with various cancer-related pathways including cell cycle, carbon metabolism, and peroxisome, which was consistent with previous studies [10, 33].

In light of its critical role in the pathogenesis of liver cancer, GADD45G may be a valuable target for anti-cancer drugs. Natural products have received increasing attention in the treatment of malignant cancer patients due to their advantages of low side effects and wide range of biological activities. 4MOD belongs to a new flavonoid monomer with anti-inflammatory, anti-allergic, and anti-tumor activities [23, 34]. Park et al. demonstrated that 4MOD inhibited osteosarcoma cell growth and promoted cell apoptosis via inhibiting the activation of JAK2/STAT3 pathway [35]. Li et al. also confirmed that 4MOD could inhibit the growth of astroglioma cells U87 through in vivo and in vitro experiments [23]. In our previously study, we conducted in vitro experiment and showed that 4MOD had significant inhibitory effects on HCC cells by regulating GADD45G expression [15]. However, the anti-liver cancer effects and mechanism of 4MOD in vivo required further investigation.

In this study, xenograft nude mouse model was used to ascertain the suppression effects of 4MOD on HCC in vivo. The results demonstrated that 4MOD significantly inhibited liver cancer growth and showed no obviously cytotoxic effect on normal cells. To ascertain whether the anti-HCC effects of 4MOD treatment in vivo were associated with GADD45G upregulated expression, we used qRT-PCR and IHC assays to compare GADD45G mRNA and protein expression in xenograft tissues with or without 4MOD treatment. As expected, the 4MOD treatment increased the expression of GADD45G at both protein and mRNA levels.

Several limitations of this study were also identified. Firstly, the associations between GADD45 mRNA expression and clinicopathological parameters, as well as with survival data, were limited in GEO database. Secondly, the sample size for IHC verification of GADD45G protein expression in HCC and non-HCC tissues was small, and future studies with more HCC samples are needed to ensure the reliability of results. Thirdly, further experiments are also needed to explore the exact relationship between GADD45G and 4MOD. Eventually, the exact function and molecular mechanism of GADD45G in liver cancer require further exploration.

## Conclusions

To sum up, this study indicates that GADD45G acts as a tumor suppressor gene in HCC. GADD45G is downregulated in HCC and predicts poor prognosis of HCC patients, suggesting that it may be a valuable biomarker for diagnosis, prognosis, and treatment of HCC patients. Moreover, this study confirms that 4MOD inhibits the growth of liver cancer in vivo through upregulating GADD45G, which increases our understanding of the anti-tumor effect and mechanism of 4MOD in HCC.

### Supplementary Information

**Additional file 1: Fig. S1.** Histopathological examination of major organs of nude mice.

**Additional file 2: Table S1.** Relationship between GADD45G protein expression and clinicopathological parameters in HCC patients.

**Additional file 3: Table S2.** List of GO-BP category analysis of GADD45G co-expression genes.

**Additional file 4: Table S3.** List of GO-MF category analysis of GADD45G co-expression genes.

**Additional file 5: Table S4.** List of GO-CC category analysis of GADD45G co-expression genes.

**Additional file 6: Table S5.** List of KEGG category analysis of GADD45G co-expression genes.

## Data Availability

Data and materials are available at TCGA, GTEx, and GEO databases.

## Abbreviations

GADD45G: Growth arrest and DNA damage-inducible gene gamma
4MOD: 4-Methoxydalbergione
HBV: Hepatitis B virus
HCV: Hepatitis C virus
HCC: Hepatocellular carcinoma
qRT-PCR: Quantitative real-time polymerase chain reaction
IHC: Immunohistochemistry
RNA-seq: RNA sequencing
LIHC: Liver hepatocellular carcinoma
GTEx: Genotype-tissue expression project
TCGA: The cancer genome atlas
GEO: Gene expression omnibus
SMD: Standardized mean differences
SROC: Summarized receiver’s operating characteristics
AUC: Area under the curve
KM: Kaplan-Meier
OS: Overall survival
PFI: Progress free interval
DSS: Disease specific survival
aDC: Activated dendritic cells
DC: Dendritic cells
iDC: Immature DC
pDC: Plasmacytoid DC
Tcm: T central memory cells
Tem: T effector memory cells
TFH: T follicular helper cells
Tgd: T gamma delta cells
TReg: T regulatory cells
GO: Gene Ontology
KEGG: Kyoto Encyclopedia of Genes and Genomes
95% CI: 95% Confidence interval
BP: Biological process
CC: Cellular components
MF: Molecular function

## References

[1] Sung H, Ferlay J, Siegel RL, Laversanne M, Soerjomataram I, Jemal A (2021). Global cancer statistics 2020: GLOBOCAN estimates of incidence and mortality worldwide for 36 cancers in 185 countries. CA Cancer J Clin.

[2] Gilles H, Garbutt T, Landrum J (2022). Hepatocellular carcinoma. Crit Care Nurs Clin North Am.

[3] Yang JD, Hainaut P, Gores GJ, Amadou A, Plymoth A, Roberts LR (2019). A global view of hepatocellular carcinoma: trends, risk, prevention and management. Nat Rev Gastroenterol Hepatol.

[4] Sperle I, Steffen G, Leendertz SA, Sarma N, Beermann S, Thamm R (2020). Prevalence of hepatitis B, C, and D in Germany: results from a scoping review. Front Public Health.

[5] Rushing BR, Selim MI (2019). Aflatoxin B1: A review on metabolism, toxicity, occurrence in food, occupational exposure, and detoxification methods. Food Chem Toxicol.

[6] Huang DQ, Mathurin P, Cortez-Pinto H, Loomba R (2023). Global epidemiology of alcohol-associated cirrhosis and HCC: trends, projections and risk factors. Nat Rev Gastroenterol Hepatol.

[7] Plaz Torres MC, Jaffe A, Perry R, Marabotto E, Strazzabosco M, Giannini EG (2022). Diabetes medications and risk of HCC. Hepatology.

[8] Yang L, Wang Q, Cui T, Huang J, Jin H (2021). Reporting and performance of hepatocellular carcinoma risk prediction models: based on TRIPOD statement and meta-analysis. Can J Gastroenterol Hepatol.

[9] Liu Q, Gao P, Li Q, Xu C, Qu K, Zhang J (2021). Long non-coding RNA SNHG16 as a potential biomarker in hepatocellular carcinoma: a meta-analysis. Medicine (Baltimore).

[10] Salvador JM, Brown-Clay JD, Fornace AJ (2013). Gadd45 in stress signaling, cell cycle control, and apoptosis. Adv Exp Med Biol.

[11] Bahar A, Bicknell JE, Simpson DJ, Clayton RN, Farrell WE (2004). Loss of expression of the growth inhibitory gene GADD45gamma, in human pituitary adenomas, is associated with CpG island methylation. Oncogene.

[12] Na YK, Lee SM, Hong HS, Kim JB, Park JY, Kim DS (2010). Hypermethylation of growth arrest DNA-damage-inducible gene 45 in non-small cell lung cancer and its relationship with clinicopathologic features. Mol Cells.

[13] Sun L, Gong R, Wan B, Huang X, Wu C, Zhang X (2003). GADD45gamma, down-regulated in 65% hepatocellular carcinoma (HCC) from 23 Chinese patients, inhibits cell growth and induces cell cycle G2/M arrest for hepatoma Hep-G2 cell lines. Mol Biol Rep.

[14] Xu G, Zhang L, Ma A, Qian Y, Ding Q, Liu Y (2015). SIP1 is a downstream effector of GADD45G in senescence induction and growth inhibition of liver tumor cells. Oncotarget.

[15] Zeng L, Qin Y, Lu X, Fang X, Huang J, Yu C (2023). 4-Methoxydalbergione elicits anticancer effects by upregulation of GADD45G in human liver cancer cells. J Healthc Eng.

[16] Zhang L, Yang Z, Ma A, Qu Y, Xia S, Xu D (2014). Growth arrest and DNA damage 45G down-regulation contributes to Janus kinase/signal transducer and activator of transcription 3 activation and cellular senescence evasion in hepatocellular carcinoma. Hepatology.

[17] Ou DL, Shyue SK, Lin LI, Feng ZR, Liou JY, Fan HH (2015). Growth arrest DNA damage-inducible gene 45 gamma expression as a prognostic and predictive biomarker in hepatocellular carcinoma. Oncotarget.

[18] Wang SY, Feng LY, Meng ZQ (2015). Bicluster and pathway enrichment analysis related to tumor progression of hepatocellular carcinoma. Eur Rev Med Pharmacol Sci.

[19] Yang YH, Mao JW, Tan XL (2020). Research progress on the source, production, and anti-cancer mechanisms of paclitaxel. Chin J Nat Med.

[20] Ruan Q, Patel G, Wang J, Luo E, Zhou W, Sieniawska E (2021). Current advances of endophytes as a platform for production of anti-cancer drug camptothecin. Food Chem Toxicol.

[21] Pfister C, Gravis G, Fléchon A, Soulié M, Guy L, Laguerre B (2021). Randomized phase III trial of dose-dense methotrexate, vinblastine, doxorubicin, and cisplatin, or gemcitabine and cisplatin as perioperative chemotherapy for patients with muscle-invasive bladder cancer. Analysis of the GETUG/AFU V05 VESPER trial secondary endpoints: chemotherapy toxicity and pathological responses. Eur Urol.

[22] Li M, Xiao Y, Liu P, Wei L, Zhang T, Xiang Z (2023). 4-Methoxydalbergione inhibits esophageal carcinoma cell proliferation and migration by inactivating NF-κB. Oncol Rep.

[23] Li R, Xu CQ, Shen JX, Ren QY, Chen DL, Lin MJ (2021). 4-Methoxydalbergione is a potent inhibitor of human astroglioma U87 cells in vitro and in vivo. Acta Pharmacol Sin.

[24] Du H, Tao T, Xu S, Xu C, Li S, Su Q (2021). 4-Methoxydalbergione inhibits bladder cancer cell growth via inducing autophagy and inhibiting Akt/ERK signaling pathway. Front Mol Biosci.

[25] Zhang R, Mo WJ, Huang LS, Chen JT, Wu WZ, He WY (2021). Identifying the prognostic risk factors of synaptojanin 2 and its underlying perturbations pathways in hepatocellular carcinoma. Bioengineered.

[26] He FY, Chen G, He RQ, Huang ZG, Li JD, Wu WZ (2022). Expression of IER3 in hepatocellular carcinoma: clinicopathology, prognosis, and potential regulatory pathways. PeerJ.

[27] Bindea G, Mlecnik B, Tosolini M, Kirilovsky A, Waldner M, Obenauf AC (2013). Spatiotemporal dynamics of intratumoral immune cells reveal the immune landscape in human cancer. Immunity.

[28] Fornace AJ, Alamo I, Hollander MC (1988). DNA damage-inducible transcripts in mammalian cells. Proc Natl Acad Sci U S A.

[29] Fornace AJ, Nebert DW, Hollander MC, Luethy JD, Papathanasiou M, Fargnoli J (1989). Mammalian genes coordinately regulated by growth arrest signals and DNA-damaging agents. Mol Cell Biol.

[30] Li T, Xu L, Teng J, Ma Y, Liu W, Wang Y (2020). GADD45G interacts with E-cadherin to suppress the migration and invasion of esophageal squamous cell carcinoma. Dig Dis Sci.

[31] Guo D, Zhao Y, Wang N, You N, Zhu W, Zhang P (2021). GADD45g acts as a novel tumor suppressor, and its activation suggests new combination regimens for the treatment of AML. Blood.

[32] Zhang X, Li Y, Ji J, Wang X, Zhang M, Li X (2021). Gadd45g initiates embryonic stem cell differentiation and inhibits breast cell carcinogenesis. Cell Death Discov.

[33] Vairapandi M, Balliet AG, Hoffman B, Liebermann DA (2002). GADD45b and GADD45g are cdc2/cyclinB1 kinase inhibitors with a role in S and G2/M cell cycle checkpoints induced by genotoxic stress. J Cell Physiol.

[34] Chan SC, Chang YS, Wang JP, Chen SC, Kuo SC (1998). Three new flavonoids and antiallergic, anti-inflammatory constituents from the heartwood of Dalbergia odorifera. Planta Med.

[35] Park KR, Yun HM, Quang TH, Oh H, Lee DS, Auh QS (2016). 4-Methoxydalbergione suppresses growth and induces apoptosis in human osteosarcoma cells in vitro and in vivo xenograft model through down-regulation of the JAK2/STAT3 pathway. Oncotarget.

