# Supplementary figures and images for: Identify GADD45G as a potential target of 4-methoxydalbergione in treatment of liver cancer: bioinformatics analysis and in vivo experiment
Source: World J Surg Oncol. 2023 Oct 13;21:324. doi: 10.1186/s12957-023-03214-3 (PMC10571512; doi:10.1186/s12957-023-03214-3)

**Fig. S1** Histopathological examination of major organs of nude mice


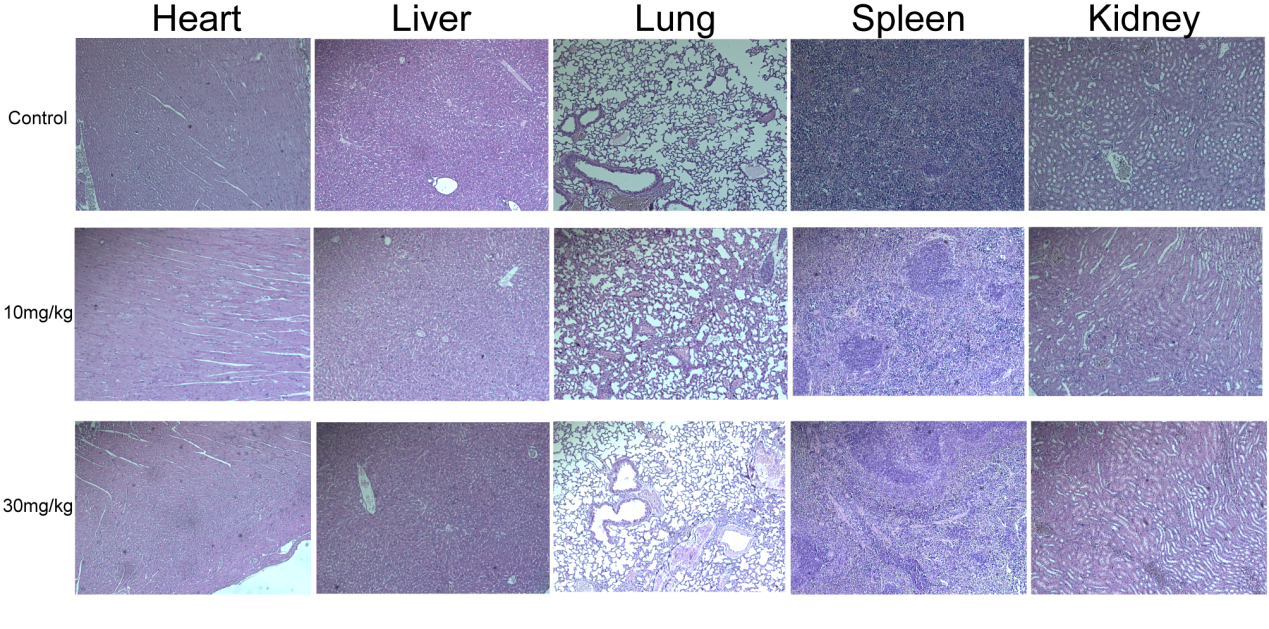

Supplement: Supplementary file 1 — Additional file 1: Fig. S1. Histopathological examination of major organs of nude mice. [file 12957_2023_3214_MOESM1_ESM.docx]
